# Use of Concept Mapping to Identify Expectations of Pharmacy Students Selecting Elective Courses

**DOI:** 10.3390/pharmacy9010014

**Published:** 2021-01-08

**Authors:** Ruth Vinall, Peter Balan

**Affiliations:** 1 College of Pharmacy, California Northstate University, Elk Grove, CA 95757, USA; 2 UniSA Business School, University of South Australia, Adelaide, SA 5001, Australia; Peter.Balan@unisa.edu.au

**Keywords:** student expectations, elective courses, delivery format, online classes, concept mapping

## Abstract

The objective of this study was to demonstrate the use of concept mapping as a method for analyzing pharmacy students’ qualitative perceptions of their expectations of elective courses and to thus help guide delivery methods and course content. A survey containing demographic, Likert scale, and open-ended questions was administered to second-year pharmacy students prior to the start of elective courses and an innovative methodology, concept mapping, was used to identify major themes relating to student expectations. The association between preferred class delivery method (online versus in person) with student gender and English-as-a-second-language status (ESL) was also assessed. Note that this study was conducted prior to the COVID-19 pandemic. Ninety-eight out of 133 students (74%) completed the survey. Overall, 56% students stated that they preferred online delivery of courses (68% of these students were female, 36% were male). ESL status did not impact preference. The most common themes relating to student course expectations were the desire to learn about the elective course topic as well “real-world” utility. Our combined data indicate that delivery method is a key factor contributing to students’ choice of elective course and that concept mapping is an effective and efficient way to help identify student expectations of elective courses.

## 1. Introduction

As per US Accreditation Council for Pharmacy Education (ACPE) guidelines, pharmacy curricula must contain didactic elective course opportunities for students which relate to general curricular areas: biomedical sciences; pharmaceutical sciences; social, administrative, and behavioral sciences; clinical sciences; community engagement; pharmacy education; and professional practice (https://www.acpe-accredit.org/). As might be expected, based on this broad guideline US pharmacy schools collectively offer a diverse range of didactic elective courses, e.g., complementary medicine, health informatics, global health, research, and oncology [1,2,3,4,5,6,7]. Typically, course topic, content, and delivery method for each didactic elective course is at the discretion of the faculty member(s) teaching the course and is based on their (or their institution’s) perceived needs and goals of the students, and/or personal preference and interests [8].

While multiple studies have demonstrated that particular electives improve student performance in the given subject area [9,10,11], and some have assessed end-of-course student satisfaction [12,13,14], to our knowledge, a study which identifies pharmacy students’ expectations of their pharmacy elective courses prior to taking them has not been conducted. Pharmacy student preference for a delivery method of didactic elective courses has also not been assessed. The goal of this study was to identify students’ course expectations at the start of didactic pharmacy elective courses. The identification of students’ course expectations is important because this information can be used to leverage and support intrinsic and/or extrinsic motivations and thereby help improve student engagement and student learning outcomes. When appropriate, this information can also be used to address and manage unrealistic and/or misguided expectations, a factor which can also lead to a lack of engagement [15]. The need to promote student engagement is a common theme throughout the ACPE 2016 accreditation guidelines (https://www.acpe-accredit.org/pharmd-program-accreditation/) and it is well accepted that student engagement, including pharmacy student engagement, is associated with improved learning outcomes, and that a lack of engagement can contribute to academic failure [10,16,17,18,19,20]. Increasing a sense of ownership through soliciting student input can help increase engagement [21,22]. Determination of student preference for delivery method is also important as an increasing number of pharmacy faculties are considering delivering courses in an online format. Several studies indicate that understanding and managing student expectations as well as ensuring student engagement may be even more important and challenging for online courses [23,24,25,26].

An innovative method for analyzing qualitative data, concept mapping, was used in this study to identify key “themes” in the data comprising student statements that were generated by free response survey questions. Including free response questions in educational research studies can provide unexpected and important insights but is often avoided due to concerns regarding the time and expertise needed to analyze and interpret the qualitative data generated. Key attributes of concept mapping include that it is a relatively quick method to implement, it produces graphical output that identifies themes and is easy to explain and understand, the graphical output shows relationships between themes, it can produce meaningful data from both small and large data sets (from 20 qualitative statements through to 120 or more), and the software is inexpensive and relatively easy to use (e.g., http://www.analytictech.com/ucinet) [27,28]. These features allow for data to be interpreted and shared with both students and administrators in a timely, understandable, and meaningful way.

## 2. Materials and Methods

### 2.1. Research Participants

Quantitative and qualitative data for this research study was obtained from second year PharmD students enrolled in one of four elective courses at California Northstate University College of Pharmacy (CNUCOP); “Clinical pharmacology”, “Drug discovery and development”, “Pharmacists in public health”, and “Special populations pharmacotherapy”. This study was approved by the CNU IRB committee (protocol #1113-01-42).

### 2.2. Data Collection

All second-year students were invited to participate in the study. A short questionnaire including demographic, Likert scale, and open-ended questions was administered prior to the start of elective courses (Table 1). Responses were voluntary, anonymous, and in writing.

### 2.3. Qualitative Data Analyses

Concept mapping was used to identify major themes describing student expectations in these courses. Qualitative data were obtained by students responding to the free response question “what are your learning expectations for the elective that you chose?”, and individual responses ranged from a few words to several sentences.

The purpose of the study was to understand responses provided by students enrolled in the same program and in the same cohort, but in separate classes. There was some variation in the proportions of males and females in these classes as well as in the proportions of students speaking English as a first language, but these variations were expected on account of the law of small numbers. As no hypothesis was formed regarding interclass variation, the pooling of comments for all four classes was warranted for the purpose of this study [29].

The combined qualitative dataset was analyzed using concept mapping. This can be described as a mixed-methods approach [30], or as a “structured methodology” [31] that “integrates qualitative individual and group processes with multivariate statistical analyses” to create a map that shows the relationships between the data [32] and reveals the key themes in a pictorial representation [33]. This is a form of social network analysis that is based on grouping individual statements using their similarities to create clusters for analysis and interpretation. This method allows relatively quick analysis of large qualitative datasets, provides a complete audit trail with a clear linkage between final output and the original source documents, and, importantly, enables ready collaboration between researchers [27,28]. The research findings are considered to be faithful to the data collected, and are regarded as trustworthy and, hence, reliable (Lincoln and Guba cited in [34]). Concept mapping is considered to be a qualitative research method that achieves reliability and validity and has gained increasing acceptance as a research method [33].

Data preparation was carried out by one researcher extracting statements from the questionnaires, and then coding these statements for their semantic similarities without taking account of their nature or their potential interpretation. This was a “bottom-up” or “grounded” approach where the researcher was not required to apply preconceived notions of the likely nodes, categories or themes, unlike coding methods that are required for the implementation of NVivo [27]. In effect concept mapping is an efficient method for carrying out incident-to-incident coding [35] that is traditionally performed using software such as NVivo, but the major difference is that statements are not deliberately interpreted into NVivo nodes but simply matched for their semantic similarity [36].

**Figure 1:**
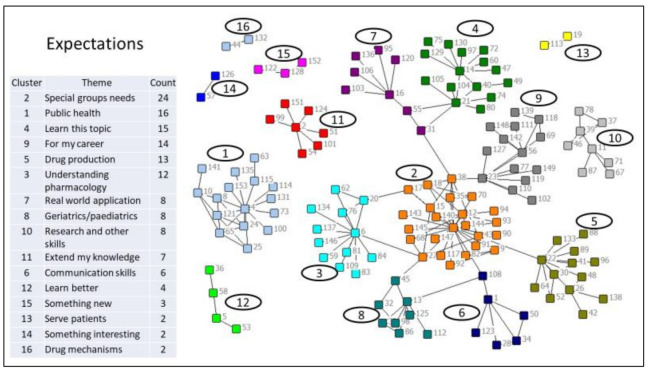
Themes describing student learning expectations.

The data coded for similarities were then analyzed using UCINET 6.0 [37] that grouped statements according to their similarities. The grouping of statements was checked by having the second researcher examine each cluster or grouping of statements for their similarities, to confirm that statements in each cluster were as homogenous in meaning as possible. This is the equivalent process when using NVivo, for a second researcher to independently code the data to arrive (ideally) at a high level of intercoder reliability by having as many agreements in coding as possible to achieve a homogeneous grouping of comments recorded in each node, to confirm the accuracy of the analysis [36]. NETDRAW [38] was then used to produce a graphics map of the clusters or concepts (Figure 1). The resulting concept map was then analyzed for community structure using the Girvan Newman algorithm [39] to identify the configuration of the concept map (Figure 1) that produced the optimum number of clusters. The optimum result was the concept map for which this algorithm produced the Q-score closest to 1, as that signifies the map whose clusters are farthest from that which could be produced randomly [40]. However, as this is “grounded” qualitative research, the researchers also reviewed other concept maps with Q-scores around the highest score to determine whether, notwithstanding the scientifically determined optimum level, the concept maps selected for analysis represented the socially constructed perceptions as seen by the research participants [41]. The researchers concluded that the concept map with the highest Q-score and with 16 clusters was the most appropriate. Both researchers then collaborated to interpret the themes from these groups of similar statements, using an abductive approach [35], and these are the equivalent of node descriptions obtained when using NVivo. This was done using the concept map (Figure 1), and applying creative thinking [28] to give each concept a name and brief description.

This process allows significant efficiency in qualitative data analysis. For example, in a study using similar student-generated data, the first researcher manually coded 126 data elements in 40 min, and needed an additional 30 min to arrive at a satisfactory cluster map and cluster descriptions. This efficiency has allowed the first researcher to carry out similar systematic analyses of student expectations using data collected during each commencement class so that results can be used to adapt delivery approaches to better meet the expectations of students in each course. In this research, additional time was needed for collaboration, but the process was none-the-less very efficient.

In this study, because each qualitative data element was identified with a particular participant, it was possible to combine the quantitative and qualitative datasets. This allowed the researchers to analyze the key themes for each category of participants (by gender), as well as by their responses to the quantitative questions in the survey that participants had completed.

### 2.4. Quantitative Data Analyses

Chi-squared analyses assessed the association between preferred class delivery method (online versus in-person) with student gender and English as a second language status.

## 3. Results

### 3.1. Participant Characteristics

Ninety-eight out of 133 (74%) second year pharmacy students consented to participate in the research study. Overall, there were more female participants (65% female and 35% male), and approximately half of the participants stated English was their second language (ESL students, 48%) (Table 2). Note that the percentage of females/males was 58.4%/41.1% in the student population from which this cohort was derived. This indicates that more females compared to males participated in the study.

### 3.2. Elective Class Delivery Preferences

Approximately half (56%) of students stated that they preferred online delivery (Table 2). As might be expected, fewer students who took the two in-person courses (Electives A and B) stated that they preferred online delivery (25% and 35% of students, respectively), while more students who took the hybrid course (Elective C) and online course (Elective D) stated that they preferred online delivery (87% and 88%, respectively). It is noteworthy that the mismatch between their course delivery preference versus the type of course they chose to take was more pronounced for the in-person courses indicating that course topic may have been more important to these students compared to delivery method. These data also indicate that online and/or hybrid delivery of these courses could be considered. More females compared to males stated that they preferred online delivery (41/60 females (68%) vs 12/33 males (36%)), and this finding was statistically significant; Chi-square statistic with Yates correction is 7.621, *p* = 0.006. A statistically significant difference between ESL and non-ESL students was not observed; Chi-square statistic with Yates correction is 2.610, *p* = 0.106. Fifty-four out of 98 students (55%) stated they felt “very strongly” or “strongly” that delivery method was important. Of these students, 35/53 (66%) felt “very strongly” or “strongly” that online delivery was important while 19/45 felt “very strongly” or “strongly” that in-person delivery was important (42%). This was a statistically significant difference (Chi-square statistic with Yates correction is 4.658, *p* = 0.031) indicating that students who preferred online delivery felt more strongly about course delivery method compared to students who preferred in-person delivery.

Subgroup analysis determined that no difference in how strongly males versus females felt about course delivery method with 21/35 (60%) males and 33/65 (51%) stating that they felt “very strongly” or “strongly” that this was important (Chi-square statistic with Yates correction is 0.991, *p* = 0.320). No difference was observed between ESL and non-ESL students either (29/46 (63%) of ESL students compared to 26/52 (50%), Chi-square statistic with Yates correction is 0.194, *p* = 0.274).

### 3.3. Student Learning Expectations

Analysis of free response data for all participants (which was generated in response to the “What are your expectations for this elective?” question) produced the cluster map shown in Figure 1. An optimum number of 16 clusters was identified [40]. Representative responses for each cluster (or “theme”) are shown in Table 2.

Themes associated with each cluster are shown in Table 3 in decreasing order of the number of comments included in each cluster (the number of comments is considered an indication of the importance of these themes). Typical comments (verbatim) for each of the identified themes are also listed in Table 3.

Many of the most prevalent themes related directly to the elective course topics; “special populations” (ranked first, and directly relates to the “Special populations pharmacology” elective course), “public health” (ranked second, and directly relates to the “Pharmacists and public health” elective course), “drug discovery and development” (ranked fifth, and directly relates to the “Drug discovery and development” elective course), “understanding pharmacology” (ranked sixth, and directly relates to both the “Clinical pharmacology and special populations pharmacology” elective courses), and “learn this topic” (ranked third and directly relates to all the elective courses). Non-course specific themes also emerged including “for my career”, “real world application”, “research skills”, “extend my knowledge”, “communication skills”, “learn better”, “something new”, “serve patients”, “something interesting”, “drug mechanisms”.

### 3.4. Analyses of Expectations by Particular Elective

The number of comments by participants enrolled in each of the different elective courses was relatively small and as such it was deemed inappropriate to conduct concept mapping-based statistical analyses for each of these, however, a noteworthy trend is that the “for my career” theme predominated in elective B (the “Special populations pharmacotherapy” elective course).

## 4. Discussion

To our knowledge, this is the first study to identify pharmacy student expectations of didactic elective courses. We demonstrate that delivery method is very important to many students, and that the primary expectation of most students is to learn more about the elective topics. Other frequently expressed expectations related to gaining real-world knowledge and to support their career. Some students also stated they expected to learn something new, to improve study and communication skills, and/or to learn something interesting. It is noteworthy that we also employ an innovative method, concept mapping, to analyze and present the qualitative data in a way that is easy to understand and interpret.

An increasing number of colleges are now offering at least some courses in an online format (https://educationdata.org/online-education-statistics/), and more students, including local students, are enrolling in these online courses [42]. In alignment with the latter, in our study we found that more students had a strong preference for online delivery of didactic elective courses compared to those who had a strong preference for in-person, although it is noteworthy that the numbers of students with a strong preference was high in both groups. Clearly delivery format is a key consideration, and our data indicate that providing didactic electives offered via both modalities would be ideal to help meet student expectations. Several studies have documented the success of online didactic elective courses for PharmD students, demonstrating that students achieved learning outcomes and were satisfied with the courses [43,44]. Porter et al. also demonstrated that there was no difference in PharmD student performance when the same didactic elective course was offered via online versus in-person delivery [44]. It is noteworthy that in our study we found that a higher percentage of female students preferred online delivery compared to male students because females typically account for more than 50% of the student body at most pharmacy schools. We did not assess why students preferred the online delivery format in our study, however, others have found that increased flexibility and the ability to self-pace learning are key factors.

Multiple studies have demonstrated that didactic elective courses can improve PharmD student competency in the subject area and are well received by students [1,2,3,4,5,6,7]. While these studies support the continued inclusion of electives in the PharmD curriculum, unlike our study, they did not assess the expectations of students. Doing so is important because mismatches in what students expect versus what faculty and administrators believe students expect (or should expect) can lead to significant frustration, loss of engagement, and/or decreased student performance [45,46]. A key expectation of students in our study was to learn more about the elective topic. While this is not entirely surprising, it is encouraging that our students placed emphasis here as it indicates that they found the elective topics that were offered of interest. Other frequently expressed student expectations related to relevance to pharmacy practice and “real-world” experiences. Many of the course-specific comments also related to the desire to be able to use the knowledge and skilled learnt. Again, perhaps this is to be expected as most students enrolled in a professional school have already set career goals and are focused on gaining knowledge and understanding to support these. In similar studies conducted in undergraduate settings, student expectations such as “enjoyment” and “easy” and “to get to know the instructor” are more prevalent and this likely reflects the fact that many of these students are not goal driven and/or may lack the maturity that is usually observed in students enrolled in professional schools. Our data indicate that faculty members should place emphasis on ensuring that their PharmD elective courses are geared towards helping prepare students for the workplace. For some faculty members, in particular non-PharmD faculty members, this may require working with internal and external stakeholders, e.g., community and hospital pharmacy managers, and pharmaceutical industry representatives, to help ensure that student expectations are adequately met. Inclusion of some of these people as guest lecturers could also be considered. In support of this, a recent study by Bullock and Horne found that more than half of students enrolled in a Clinical Skills and Business management elective course stated that their favorite aspect of the course was the inclusion of community pharmacist guest lecturers [10].

### Study Limitations

While a large proportion of the class consented to participate in this study, the study size was still relatively small (total of 98 students). Likewise, this study only included data from a single student cohort at a single college of pharmacy and as such it is not certain that similar results would be observed at other colleges of pharmacy. Further multi-site studies will be needed to validate our findings. Future studies (with larger numbers of participants) will also place focus on better understanding the linkage between the different themes by applying the process recommended by Shepherd and Suddaby [47] to develop a theory and testable model for describing student expectations in a manner that is similar to that described in Browne, Balan and Lindsay [48].

## 5. Conclusions

This study demonstrates the application of concept mapping as an effective method for analyzing qualitative data in a typical classroom situation where an educator would wish to understand student learning expectations at the beginning of a course or program delivery. This method allowed the researchers (and educators) to collaborate at each stage of data collection and analysis. In particular, this work was implemented quickly, and thus allowed the implementation of an “expectations management” approach to tailor course delivery and content to the needs of the particular classes in order to achieve improved learning outcomes. Our plan is to encourage all instructors at our institution to consider including this type of analysis. Our findings for these courses indicate that colleges of pharmacy should consider offering both online and in-person elective courses to meet the needs of pharmacy students, and that emphasis should be placed on ensuring elective courses help prepare students for practice. Addressing the needs and expectations of pharmacy students will very likely increase student engagement in elective courses and increase satisfaction levels.

## Figures and Tables

**Table 1:** Survey questions.

Question	Possible Responses
Are you male or female?	Male, Female
Is English your second language?	Yes, No
What is your preferred mode of delivery for elective courses?	Online, In person
How important was mode of delivery when choosing between the four available elective courses?	Extremely important, Very important, Quite important, Somewhat important, Unimportant
What are your learning expectations for the elective that you chose? (i.e., What do you hope to get out of the elective course?). Please try and write at least two learning expectations.	Free response

**Table 2:** Participants in this research.

	Elective A (in-Person Delivery) *n* (%)	Elective B (in-Person Delivery) *n* (%)	Elective C (Hybrid Delivery) *n* (%)	Elective D (Online Delivery) *n* (%)	Total *n* (%)
**Female**	9 (56)	23 (59)	16 (70)	14 (82)	62 (65)
**Male**	7 (44)	16 (41)	7 (30)	3 (18)	33 (35)
**English as second language**	5 (31)	21 (53)	9 (39)	11 (64)	46 (48)
**Online delivery preferred**	4 (25)	14 (35)	20 (87)	15 (88)	53 (56)

Elective A = “Drug discovery and development”, elective B = “Special populations pharmacotherapy”, elective C = “Pharmacists and public health”, elective D = “Clinical pharmacology”.

**Table 3:** Details of major themes describing student learning expectations.

Theme	Typical Comments (Verbatim) Illustrating the Theme
Special populations *	“Be more comfortable about treating or counselling special populations”; “To be more informed of special populations and less chance of making a mistake”; “I hope to gain a better understanding of how certain drugs affect certain people varying in their race, gender”.
Public health *	“Learn more about the role of pharmacists in public health”; “I hope to learn more about educating the public effectively on improving their health”; “How public health is important to pharmacists and patients”.
Drug discovery and development *	“Hope to know the progress of drug discovery”; “To understand the method of drug discovery behind medications”; “Get a better understanding of how industry works”
Understanding pharmacology *	“To gain a more profound understanding of pharmacological aspects”; “Understand more about clinical pharmacology and its usage”; “Gain knowledge in clinical pharmacology topics”
Learn this topic	“I want to learn more in depth about my elective”; “Learn the important focus point of the course”; “Just to become more of an expert in this area”.
For my career	“Be able to use what I have learned in this class for rotations and in future job”; “Learn materials that I can apply to my future career”; “Something beneficial to my profession”.
Real world application	“How to apply it in practice”; “Learn applications to real life”; “I hope to learn about how to better prepare for working in pharmacy in the real world”.
Geriatrics/pediatrics	“How to better care for children as a healthcare professional”; “How to better care for geriatrics as a healthcare professional”; “Learn more information about disease states affecting paediatric, geriatric and pregnant populations”.
Research skills	“Improve research skills”; “develop unique skills for a role in industry work”; “My expectation is to try and gain research knowledge”.
Extend my knowledge	“To give us an idea about different fields of pharmacy”; “Updated on current events relevant to course”; “Learn a new perspective in the pharmacy field”.
Communication skills	“Communication skill building”; “I want to be a better pharmacist by learning about how to approach others”; “I also want the chance to improve my public speaking skills”.
Learn better	“I also want to develop my ability to self engage in my learning and develop those skills”; “Examining myself as to how well I can learn complicated subjects are online learning and self studying”; “Also to familiarise myself with a class in an electronic setting, as this is our future due to flexibility and convenience”.
Something new	“Learn something new”; “I hope to learn more about topics not covered in typical course material”; “To learn about new research/discoveries about medications/treatments”.
Serve patients	“To better serve patients. I chose a selective because I think the information will be more useful as a pharmacist”; “How it truly is to work in a public setting as pharmacist”;
Something interesting	“Bringing interest, new fields of practice”; “Learn about interesting topics”.
Drug mechanisms	“MOA for all drugs learned”; “To understand the mechanisms of medications”.

* These themes related directly to elective topic.

## Data Availability

The data presented in this study are available on request from the corresponding author.
